# Age-related associations between heavy drinking and inconsistent condom use among unmarried adults: results from the *Brazilian National Health Survey*

**DOI:** 10.1590/0102-311XEN131924

**Published:** 2025-08-08

**Authors:** Nayara Lopes Gomes, Cláudia de Souza Lopes

**Affiliations:** 1 Instituto de Medicina Social Hesio Cordeiro, Universidade do Estado do Rio de Janeiro, Rio de Janeiro, Brasil.

**Keywords:** Condoms, Sexual Behavior, Single Person, Alcohol Drinking, Health Surveys, Preservativos, Comportamento Sexual, Pessoa Solteira, Consumo de Bebidas Alcoólicas, Inquéritos Epidemiológicos, Condones, Conducta Sexual, Persona Soltera, Consumo de Bebidas Alcohólicas, Encuestas Epidemiológicas

## Abstract

Risky sexual behaviors, such as the nonuse of condoms, are identified in the literature as a possible outcome of excessive alcohol use. However, studies on this topic are focused on the high-risk population, namely young adults, men who have sex with men, and sex workers, which limits the analysis of life stages in this association. Thus, this study aimed to verify, for the first time, the association between heavy drinking, at global-level, and inconsistent condom use among unmarried, not cohabiting Brazilians aged 18 to 59 years in various phases of adulthood. Data were obtained from the 2019 *Brazilian National Health Survey* (N = 15,835). Adjusted prevalence ratios and confidence intervals were estimated using Poisson regression models stratified by sex. Heavy drinkers, unmarried, not cohabiting adults were significantly more likely to report inconsistent condom use when compared with non-heavy drinkers for all age groups and both sexes. However, significant differences were not found for this association between the phases of adulthood.

## Resumo

A literatura identifica comportamentos sexuais de risco, como o não uso de preservativos, como um possível desfecho do uso excessivo de álcool. No entanto, os estudos sobre essa temática se voltam a populações de alto risco - adultos jovens, homens que fazem sexo com homens e profissionais do sexo -, o que limita a análise das fases da vida nessa associação. Assim, este estudo buscou verificar, pela primeira vez, a associação entre o consumo abusivo de álcool, em nível global, e o uso inconsistente de preservativo entre brasileiros solteiros não coabitantes com idade entre 18 e 59 anos em várias fases da vida adulta. Os dados foram obtidos da *Pesquisa Nacional de Saúde* de 2019 (N = 15.835). Razões de prevalência ajustadas e intervalos de confiança foram estimados por modelos de regressão de Poisson estratificados por sexo. Bebedores pesados, solteiros e não coabitantes foram significativamente mais propensos a relatar uso inconsistente de preservativo do que aqueles que não consomem álcool de forma excessiva para todas as faixas etárias e ambos os sexos. No entanto, essa associação não mostrou diferenças significativas entre as fases da vida adulta.

## Resumen

Las conductas sexuales de riesgo, como la falta de uso de condones, se identifican en la literatura como un posible resultado del consumo excesivo de alcohol. Sin embargo, los estudios sobre este tema se centran en la población de alto riesgo, es decir, los adultos jóvenes, los hombres que tienen sexo con hombres y los trabajadores sexuales, lo que limita el análisis de las etapas de la vida en esta asociación. Así, este estudio tuvo como objetivo verificar, por la primera vez, la asociación entre el consumo excesivo de alcohol, a nivel mundial, y el uso inconsistente del condón entre brasileños solteros no cohabitantes de 18 a 59 años en diversas fases de la adultez. Los datos se obtuvieron de la *Encuesta Nacional de Salud* de Brasil de 2019 (N = 15.835). Las razones de prevalencia ajustadas y los intervalos de confianza se estimaron mediante modelos de regresión de Poisson estratificados por sexo. Los consumidores excesivos de alcool, los solteros y los adultos que no cohabitaban eran significativamente más propensos a informar sobre el uso inconsistente del condón en comparación con personas que no consumen alcohol en exceso en todos los grupos de edad y en ambos sexos. Sin embargo, no se encontraron diferencias significativas para esta asociación entre las fases de la edad adulta.

## Introduction

Sexuality is an important aspect of human life. However, the way in which it is explored can lead to serious health consequences. Risky sexual behaviors are major threats to overall health, especially sexual and reproductive health, which can be compromised by issues such as sexually transmitted infections (STIs) and unintended pregnancies.

Recent studies have highlighted the increase in STIs in Brazil and other countries in the world. In 2022, for example, the highest increases in syphilis cases were observed in the Americas and African regions ^1^. In Brazil, from 2012 to 2022, there was an increase of approximately 10% and 18% in the detection rate of AIDS (per 100,000 inhabitants) in men aged 20 to 24 and 25 to 30 years, respectively ^2^. Additionally, a significant growth trend has been observed in syphilis, particularly acquired syphilis ^3^, and one of the contributing factors might be the reduction in condom use ^4^. 

Several studies have pointed to an association between alcohol use and risky sexual behaviors, such as condom nonuse or inconsistent condom use and having multiple sexual partners ^5^ ^,^ ^6^ ^,^ ^7^ ^,^ ^8^ ^,^ ^9^. However, these findings are often focused on populations more exposed to HIV or excessive alcohol consumption, such as sex workers, men who have sex with men, and/or young adults. This limits the exploration of how different life stages may affect the relationship between alcohol use and risky sexual behaviors ^10^. 

In the context of sexuality, alcohol consumption is sometimes seen as advantageous by individuals seeking effects such as feelings of relaxation and self-confidence, facilitated communication, increased pleasure, and improved sexual performance ^11^ ^,^ ^12^ ^,^ ^13^. However, another important effect of alcohol is a reduction in cognitive ability, which can alter individuals’ risk perception of contracting an STI when engaging in unprotected sexual intercourse ^14^ ^,^ ^15^ ^,^ ^16^.

According to Leigh & Stall ^17^, there are three levels of assessment of the association between alcohol use and risky sexual behaviors: (i) global-level, in which the association is evaluated via an indicator of alcohol consumption in general, such as the frequency of alcohol consumption in a given week; (ii) situational-level, in which alcohol consumption is evaluated in specific sexual contexts, such as before or during sexual intercourse; and (iii) event-level, in which the evaluation of alcohol consumption occurs during a specific sexual event, for example, in the first or last sexual intercourse.

The relationship between alcohol use and risky sexual behaviors is complex, and at all levels, the existence of a correlation can be attributed to factors such as characteristics related to the subjects’ personalities or their lifestyles ^12^ ^,^ ^18^ ^,^ ^19^, which are often disregarded in studies.

However, the association between risky sexual behavior and alcohol use found even at the global-level, especially in countries where data sources for this type of research are scarce, may suggest a causal relationship demanding further investigation.

In Brazil, as in other countries, there are few population-based studies focusing on the adult population that enable the investigation of risky sexual behaviors ^20^ ^,^ ^21^ and their relationship with alcohol use. The most recent study on this association at national level focused on adults was an exploratory study conducted in 2008 ^22^. Moreover, there are no records of population-based studies evaluating the relationship between alcohol use and risky sexual behaviors across various stages of adulthood.

Additionally, a recent study showed that the prevalence of heavy drinking among Brazilians is higher for single young and middle-aged people than for older adults. An increase in this behavior has been observed in recent years, especially among young people and females ^23^. Worldwide, a growing trend of alcohol consumption and episodic heavy drinking is forecast for the coming years ^24^, reinforcing the importance of studies related to this topic and its consequences.

This study aimed to evaluate the association between heavy drinking and inconsistent condom use at global-level among unmarried non-cohabiting adults by age group. 

## Materials and methods

### Participants and procedures

Data were obtained from the 2nd edition of the 2019 *Brazilian National Health Survey* (2019 PNS, acronym in Portuguese) conducted by the Brazilian Institute of Geography and Statistics (IBGE, acronym in Portuguese), in partnership with the Brazilian Ministry of Health.

The 2019 PNS is a nationwide household survey, and its sampling plan was a three-stage cluster design. In the first stage, the primary sampling units (PSUs) were randomly selected from census enumeration areas. In the second stage, a random selection of households within each of the PSUs was conducted. Finally, in the third and last stage, a household member aged 15 or older (called selected resident) was randomly chosen at the time of the interview to answer a broader questionnaire ^25^.

The new module of sexual activity was included for the first time in the survey and only selected residents aged 18 years or older answered the questions ^26^. For this study, only unmarried (single, divorced, or widowed) and not cohabiting with a partner individuals aged 18 to 59 years were included, assuming that, in the context of marriage, the expectations about monogamy and alternative forms of birth control are more decisive for condom nonuse.

Individuals who reported never having had sexual intercourse or not having had sexual intercourse in the previous 12 months were excluded, as well as those who refused to answer questions about sexual activity or condom use during that period. 

The 2019 PNS was approved by the Brazilian National Research Ethics Committee (process 3,529,376, dated August 23, 2019). The participants provided consent in two stages: first, at the beginning of the household interview, and second at the interview with the selected resident ^25^.

### Measures

#### Inconsistent condom use

Inconsistent condom use was investigated among individuals who reported having had sexual intercourse in the previous 12 months using the question: “In the past 12 months, during the sexual intercourse(s) you have had, how often did you use a condom?”. Individuals who answered “never” or “sometimes” were classified as inconsistent condom users.

#### Heavy drinking

To assess heavy drinking, the following questions were used: “How many days a week do you usually consume an alcoholic beverage?” and “In general, on the days you drink, how many doses of alcohol do you consume?”. In our study, heavy drinking was considered as the weekly consumption of 8 or more drinks (for females) and 15 or more drinks (for males), following the Centers for Disease Control and Prevention (CDC) criteria ^27^. 

#### Sociodemographic variables

The sociodemographic variables considered were: (i) biological sex (male and female); (ii) age groups (18 to 24, 25 to 39, and 40 to 59 years) to evaluate youth separately from other stages of adulthood; (iii) self-reported skin color or ethnicity (white, black, mixed-race, and other); (iv) schooling level (incomplete elementary school, complete elementary school, and complete high school or higher); (v) employment status (employed and unemployed); and (vi) household area (urban and rural).

### Statistical analysis

The study sample characteristics were summarized as totals and percentages according to sex and age group. The prevalence and respective 95% confidence intervals (95%CI) of heavy drinking consumption were also estimated according to sex and age group.

The analyses were performed using Stata software version 14 (https://www.stata.com) with survey commands (*svy*) used for complex survey data. The results considered the study design and sampling weights.

Poisson regression models were used to analyze the association between heavy drinking and inconsistent condom use considering that this approach provides better estimates than logistic regression when the outcome is not rare ^28^. Adjusted prevalence ratios for sociodemographic variables, and their respective 95%CI, were reported. 

Stratified models by age group and sex were estimated. However, the formal test for age groups differences in the association between heavy drinking and inconsistent condom use was performed by including an interaction term between age group and heavy drinking in non-stratified models. Multiple hypothesis testing with Bonferroni correction ^29^ were performed to jointly assess the significance of the model’s interaction coefficients. The results associated with a p-value less than 0.05 were considered statistically significant.

In addition, to evaluate possible additive interactions, the average marginal effects of heavy drinking on the predicted probability of inconsistent condom use were estimated for different age groups using the Stata margins command.

## Results

A total of 24,685 unmarried and not cohabiting with a partner residents aged 18 to 59 years were interviewed in the sexual activity module of the 2019 PNS. Of these, individuals were excluded if they reported never having had sexual intercourse (n = 1,331; 5.4%), having not had sexual intercourse in the previous 12 months (n = 6,119; 24.8%), or if they refused to answer questions about sexual intercourse or condom use (n = 1,400; 5.7%). This resulted in a final sample of 15,835 adults.

In the study sample, 22.6% were aged 18 to 24, 41.1% were 25 to 39, and 36.3% were 40 to 59, while the average age was 35 years old. The mixed-race self-reported skin color and those living in urban areas were predominant in all age groups. The prevalence of lower levels of schooling, employed individuals, and inconsistent condom use increased with participants’ age (Table 1). Inconsistent condom use was also higher among females for all age groups. 

**Table 1:** Sociodemographic characteristics and inconsistent condom use by sex and age group (years).

Characteristics	Total (N = 15,835)	Males			Females		
		18 to 24 (n = 1,995)	25 to 39 (n = 3,098)	40 to 59 (n = 2,933)	18 to 24 (n = 1,584)	25 to 39 (n = 3,412)	40 to 59 (n = 2,813)
	%	%	%	%	%	%	%
Skin color or ethnicity							
White	34.4	31.9	34.7	35.6	32.4	32.8	37.6
Black	12.6	13.6	12.8	14.3	11.4	13.4	12.7
Mixed-race	53.8	53.2	50.8	48.8	54.6	52.4	48.0
Other	1.4	1.3	1.7	1.4	1.6	1.4	1.6
Schooling level							
Incomplete elementary school	22.2	12.4	19.4	42.5	8.3	14.4	28.4
Complete elementary school	15.5	27.0	14.7	13.5	18.3	12.8	12.2
Complete high school or higher	62.3	60.6	65.9	44.0	73.4	72.8	59.4
Household area							
Urban	84.5	79.8	82.7	76.4	87.7	89.2	90.4
Rural	15.6	20.2	17.3	23.6	12.2	10.8	9.6
Employment status							
Employed	76.2	67.6	84.9	84.0	55.3	76.3	76.6
Unemployed	23.8	32.4	15.1	16.1	44.7	23.7	23.4
Inconsistent condom use							
No	50.2	61.2	56.5	54.0	46.1	43.1	42.5
Yes	49.8	38.7	43.4	46.0	53.9	56.9	57.4

**Figure 1:**
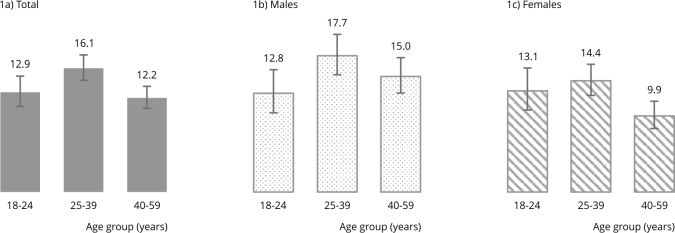
Prevalence of heavy drinking (%) among sexually active unmarried adults.

The prevalence of heavy drinking in the unmarried and not cohabiting with a partner population was 13.9%. No significant difference in heavy drinking was observed between individuals aged 18 to 24 years and middle-aged individuals (25 to 39 and 40 to 59 years), as shown in Figure 1. Among males, no significant differences in heavy drinking consumption (weekly consumption of 15 or more alcoholic drinks) were observed across the age groups. However, among females, the prevalence of heavy drinking (weekly consumption of 8 or more alcoholic drinks) was significantly lower among those aged 40 to 59 years (9.9%) compared with those aged 25 to 39 years (14.4%). 

Heavy drinking prevalence was similar between males and females aged 18 to 24 and 25 to 39 years. However, among those aged 40 to 59 years, the prevalence was significantly higher among males (15%) than among females (9.9%) (Figure 1).

Adjusted prevalence ratios for the association between heavy drinking and inconsistent condom use were significant for all strata analyzed (Table 2). The results refer to the models stratified by age groups and sex.

**Table 2:** Associations between heavy drinking and inconsistent condom use.

Sex/Predictor	Age group (years)					
	18 to 24		25 to 39		40 to 59	
	Adjusted PR	95%CI	Adjusted PR	95%CI	Adjusted PR	95%CI
Total						
Heavy drinking	1.32	1.14; 1.54	1.31	1.19; 1.44	1.26	1.13; 1.40
Heavy drinking x age group interaction *	F(2,7449) = 0.17; p = 0.841					
Female						
Heavy drinking	1.29	1.08; 1.53	1.16	1.04; 1.30	1.21	1.06; 1.37
Heavy drinking x age group interaction *	F(2,7351) = 0.37; p = 0.689					
Male						
Heavy drinking	1.40	1.10; 1.78	1.49	1.26; 1.75	1.31	1.11; 1.54
Heavy drinking x age group interaction *	F(2,7434) = 0.54; p = 0.580					

95%CI: 95% confidence interval; PR: prevalence ratio.

Note: models adjusted for sex, skin color or ethnicity, employment status, schooling level, household situation, and heavy drinking, considering not heavy drinkers as the basic category. Statistically significant estimates are presented in bold.

* Multiple hypothesis testing with Bonferroni correction results to evaluate the statistical significance of the interaction between heavy drinking variable and the age groups included in full models with the same variables mentioned in the note above in addition to the age group variable and the interaction with heavy drinking.

Nevertheless, the interaction between age groups and heavy drinking was not significant [F(2,7449) = 0.17, p = 0.841] indicating that there is no evidence of differences in this association across age groups. The same was observed for both sexes.

The adjusted prevalence ratio for the association between heavy drinking and inconsistent condom use was 1.32 (95%CI: 1.14; 1.54) among young adults (18 to 24 years) and 1.31 (95%CI: 1.19; 1.44) among those aged 25 to 39 years. Among individuals aged 40 to 59 years, it was 1.26 (95%CI: 1.13; 1.40). 

**Figure 2:**
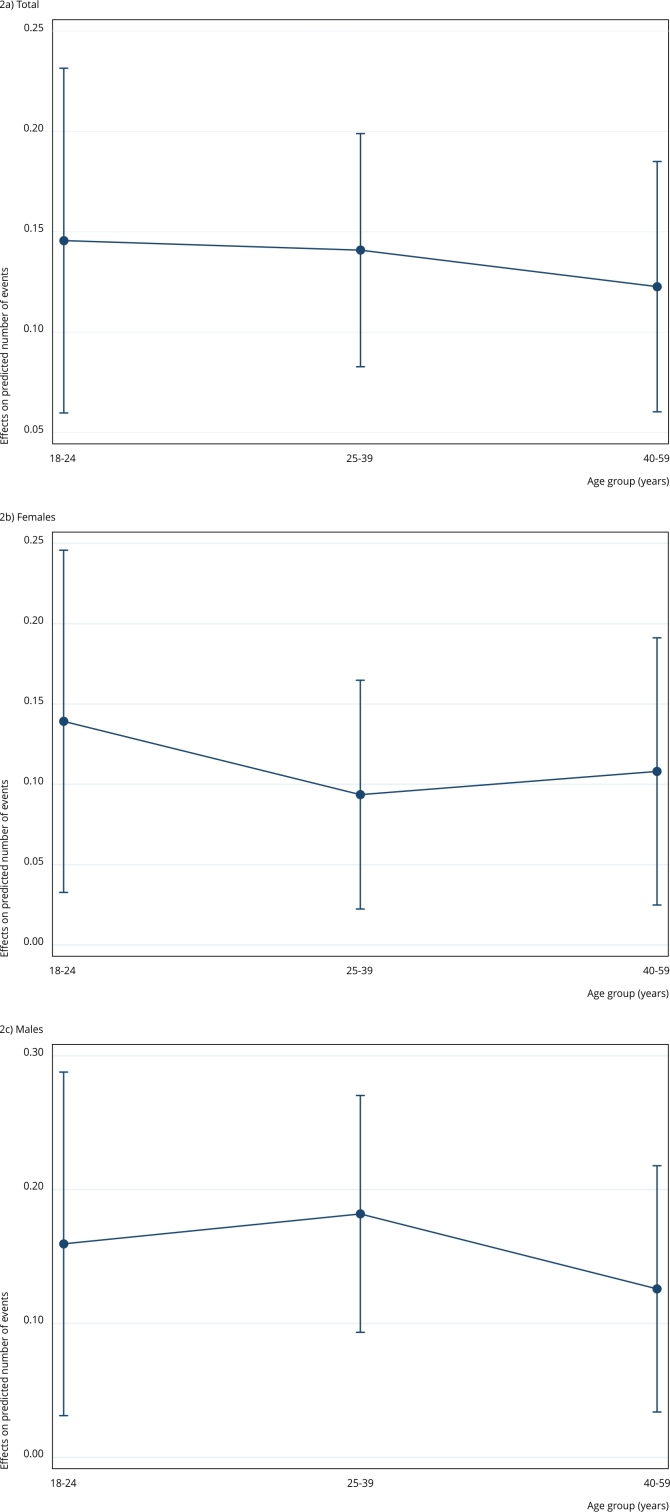
Mean marginal effects of heavy drinking on inconsistent condom use by age group.

Considering the results of the additive interactions, that is, when evaluating the average marginal effects of heavy drinking according to age groups, an increase of nearly 15% (95%CI: 5.98; 23.14) in the probability of inconsistent condom use was observed among young adults (18 to 24 years) who present a heavy drinking pattern compared to others (Figure 2). Among individuals aged 25 to 39 years, the increase was around 14% (95%CI: 8.28; 19.89), whereas those aged 40 to 59 showed an increase of about 12% (95%CI: 6.00; 18.50).

## Discussion

Our findings suggest that the prevalence of heavy drinking among Brazilian unmarried and not cohabiting with a partner is substantial, not only among young adults (18 to 24 years), who are frequently the target in the existing literature, but also among other middle-aged groups. This pattern aligns with broader social and cultural shifts in Brazil, in which delayed marriage, extended singlehood, and evolving social norms surrounding alcohol consumption have reshaped lifestyle behaviors. Given that alcohol plays a significant role in social interactions, it is likely that unmarried and not cohabiting with a partner individuals rely on drinking as a means of social engagement, which may explain the sustained high prevalence across different age groups. Additionally, a recent study by Plens et al. ^30^ found that Brazilian unmarried and not cohabiting with a partner adults are twice as likely to engage in heavy drinking compared to their married counterparts, further reinforcing the association between socialization patterns and alcohol use. 

We found that heavy drinking is associated with inconsistent condom use among Brazilian unmarried and not cohabiting with a partner adults across different age groups. However, the magnitude of this relationship was not statistically significant between young and middle-aged adults. 

Most studies investigating the link between alcohol consumption and condom use among adults focus on the role of the partner or biological sex in the relationship ^31^ ^,^ ^32^ ^,^ ^33^ ^,^ ^34^. Research exploring age-related interactions is limited and, among the existing studies, only specific age groups of adults (such as young or middle-aged adults) are included as target populations ^10^ ^,^ ^35^ ^,^ ^36^.

For example, Swartzendruber et al. ^35^, found that the number of drinks consumed per month was associated with condom nonuse among African females aged 14 to 24 years old with an increased chance from the age 22.

In one of the rare population-based studies on this topic, conducted in southern Africa and focused on young and middle-aged adults, researchers identified significant event-level associations. The study found that individuals who were drunk during their last sexual encounter were more likely to have unprotected sex. Among females, this association was marginal, and no age-specific results were reported ^32^. 

Similarly, Guo et al. ^34^ found that unmarried Chinese individuals aged 15 to 24 who consumed alcohol or smoked cigarettes were more likely to engage in unprotected sex. This finding aligns with our study, although the authors observed a stronger association among females, whereas our results indicate higher prevalence ratios among males, particularly those aged 25 to 39.

In Brazil, the only study to investigate the relationship between alcohol and condom use among adults at the national level was an exploratory study based on data from a probabilistic sample of 5,040 respondents aged 16 to 65 years living in large urban regions of Brazil in 2005. A less frequent condom use at the last sexual intercourse was observed among young and middle-aged individuals, and young males in stable relationships and who reported regular alcohol use and/or illicit drug use ^22^. No relevant differences were reported among individuals in casual relationships. However, an important limitation of that study was that alcohol consumption was assessed over the life course and in conjunction with the use of illicit drugs.

Other Brazilian studies on this topic have primarily focused on young adults, university students, or high school students. Despite these demographic limitations, there is a consistent body of evidence supporting the association between alcohol consumption and unprotected sex ^37^ ^,^ ^38^ ^,^ ^39^ ^,^ ^40^ ^,^ ^41^.

Although our study used global measures of alcohol consumption, existing evidence suggests that overall drinking patterns correlate with situational drinking behaviors among adult males and females ^10^. This indicates that the observed association between heavy drinking and inconsistent condom use may also present at other levels of evaluation, though Brazilian studies have yet to explore this in depth.

Given that both heavy drinking and inconsistent condom use constitute significant public health concerns, the findings of this study should be integrated into ongoing public health initiatives and campaigns aimed at addressing alcohol consumption and sexual risk behaviors. The observed association between heavy drinking and inconsistent condom use across all age groups underscores a critical public health issue, as it is a well-established risk factor for STIs and unintended pregnancies, both of which remain highly prevalent in Brazil ^2^ ^,^ ^3^ ^,^ ^42^. These results highlight the necessity of targeted interventions that incorporate discussions on alcohol use and sexual health, particularly within harm reduction strategies and comprehensive sexual health education programs aimed at basic education (children and adolescents).

In this context, Brazil has implemented several harm reduction programs for alcohol consumption ^43^, including initiatives led by the Brazilian Ministry of Health and the Health and Prevention in Schools Project. This project is part of the broader Health at School Program, and focuses on younger populations, particularly children and adolescents in basic education ^44^ ^,^ ^45^. However, our findings indicate that middle-aged adults also engage in high-risk behaviors, highlighting a potential gap in these initiatives. The study raises the question of whether current strategies effectively address older age groups, particularly unmarried and not cohabiting with a partner individuals, or whether adjustments are necessary to ensure their effectiveness across different life stages.

Additionally, considering the strong association between alcohol consumption and unprotected sex, it is important to examine how these behaviors intersect with other social determinants of health, such as income level, employment status, and schooling. Future research and policy planning should incorporate these findings into comprehensive sexual health programs, ensuring that vulnerable populations receive tailored interventions that address their specific needs.

Beyond STI risk and unintended pregnancies, the interaction between alcohol use and sexual behavior carries broader health implications ^19^ ^,^ ^36^. Heavy drinking is associated with a range of adverse health outcomes, including liver disease, cardiovascular issues, and mental health disorders such as depression and anxiety ^46^ - conditions that disproportionately impact Brazilian adults. The compounded risk of engaging in unsafe sexual practices further exacerbates these health burdens, necessitating integrated healthcare approaches that address both substance use and sexual health.

### Strengths and limitations of the study

A major strength of this study is its use of nationally representative data, enabling generalizable conclusions about the Brazilian adult population. Unlike previous research, which often focused on youth or specific regions, this study provides a broader perspective on risk behaviors across different life stages. Additionally, the statistical modeling approach enhances the robustness of the findings, offering valuable insights into the association between alcohol use and sexual risk-taking. Most studies conducted on this topic in Brazil are descriptive, referring to specific regions of the country or aimed at younger populations, and they do not allow comparisons across the different life stages ^22^ ^,^ ^37^ ^,^ ^38^ ^,^ ^39^ ^,^ ^40^ ^,^ ^41^.

However, some limitations should be considered. The reliance on self-reported data introduces the potential for recall and social desirability bias ^47^ ^,^ ^48^. Because the question about condom use was based on the past 12 months, participants may not accurately recall all episodes of condom use or non-use. This may lead to under- or over-reporting of behaviors, which could hinder the accuracy of the data. Similarly, the question regarding binge drinking may be impacted by recall bias, as participants may have difficulty recalling the exact number of days they drank, or the number of drinks consumed on each of those days ^49^. This may lead to under- or over-reporting of consumption. Moreover, individuals who were under the influence of excessive alcohol consumption before sexual intercourse may have additional difficulty remembering whether they used a condom. Social desirability bias should also be considered, particularly given the sensitive nature of the topics examined. Due to social expectations, individuals may underreport alcohol consumption or overreport condom use. 

Another limitation concerns the measurement of alcohol consumption. The study employed global-level alcohol consumption data rather than situational or event-level data, which would more precisely capture drinking behavior during specific sexual encounters. This limitation suggests the need for future studies incorporating more nuanced measures of alcohol consumption to better understand its direct impact on sexual decision-making. 

Non-response bias should also be considered. In our sample of 24,685 unmarried and not cohabiting with a partner residents aged 18 to 59 who were interviewed in the sexual activity module of the 2019 PNS, 5.7% did not respond to questions about sexual intercourse or condom use. These individuals may exhibit different behaviors from respondents - potentially engaging in less healthy lifestyles. Consequently, this bias is likely to result in an underestimation of the associations under investigation.

Additionally, relevant confounding variables in this relationship, such as lifestyle and the individual’s propensity for risk-taking and partner type, were not measured nor included in the models. 

Lastly, the data used in this study did not account for partner-related factors, which may play a crucial role in condom use decisions. Variables such as partner drinking behavior, relationship status, and power dynamics in sexual negotiations could further elucidate the association between alcohol use and sexual risk-taking. Including these factors in future research could provide a more comprehensive understanding of the interplay between individual and relational influences on sexual health.

## Conclusion

This study represents the first nationwide investigation in Brazil examining the relationship between heavy alcohol consumption and condom use patterns across different age cohorts within the unmarried and not cohabiting with a partner adult population. The findings demonstrate that heavy alcohol consumption was significantly associated with reduced condom use across all age groups and both sexes analyzed.

These results underscore the critical importance of examining alcohol-condom use associations across different demographic segments of the adult population. The findings provide valuable directions for future research investigating the potential causal role of hazardous alcohol consumption in condom non-adherence. Additionally, these results highlight the necessity of incorporating alcohol-related considerations into public health policies and prevention campaigns, particularly those targeting unmarried, not cohabiting young and middle-aged adults, among whom heavy drinking prevalence was notably elevated.
